# A Novel LHX3 Variant Associated With Combined Pituitary Hormone Deficiency in an Indian Kindred

**DOI:** 10.7759/cureus.111778

**Published:** 2026-06-29

**Authors:** Sreenivasan Palaniappan, Subbiah Sridhar, Senthilkumar Thasarathan, Dhivya Shanmugam, Pinaki Dutta

**Affiliations:** 1 Endocrinology, Madurai Medical College, Madurai, IND; 2 Endocrinology, G. Kuppuswamy Naidu Memorial Hospital (GKNMH), Coimbatore, IND; 3 Scientific Affairs, MedGenome Labs, Bangalore, IND; 4 Endocrinology, Post Graduate Institute of Medical Education and Research (PGIMER), Chandigarh, IND

**Keywords:** combined pituitary hormonal deficiency, hypopituitarism, lhx3, multiple pituitary hormone deficiency, whole exome sequencing

## Abstract

LHX3, a LIM-homeodomain transcription factor, is crucial in pituitary development. Mutations of LHX3 are infrequently described in the Indian subcontinent. We report two siblings born of consanguineous marriage with combined pituitary hormone deficiency (CPHD) who presented with short stature and extra-pituitary skeletal abnormalities. Whole-exome sequencing (WES) of both siblings revealed an identical novel in-frame deletion variant in the LHX3 gene c.634_636del (p.Glu212del). Both parents of the patients were heterozygous carriers of the same LHX3 variant. The genetic sequencing data did not reveal any other potentially causal variants of other candidate genes that could be associated with CPHD. Furthermore, the conservation of the reference region across species and the expression of LHX3 in pituitary tissue provide additional evidence supporting its pathogenic role. This in-frame deletion, according to theoretical models, could potentially disrupt protein-protein interactions, DNA binding, and protein stability, ultimately disrupting the specification of pituitary cell types. Both patients exhibited a remarkable response to recombinant growth hormone therapy. This novel variant provides a foundation for future functional studies and contributes to the expanding genetic spectrum of LHX3 variants in CPHD.

## Introduction

Combined pituitary hormone deficiency (CPHD) is a clinical condition characterized by partial or complete loss of two or more hormones secreted from the pituitary gland [1]. The prevalence of CPHD is around one in 8,000 individuals worldwide [1]. Recent advances in understanding pituitary gland development have provided novel insights into the etiological and genetic basis of CPHD. Currently, 33 genes have been reported to be associated with CPHD [2]. Overall, PROP1 is the widely reported gene followed by POU1F1, HESX1, LHX4, LHX3, OTX2, GLI2, and SOX2 [3]. Recent advances have greatly expanded our understanding of the genetic basis of CPHD. The identification of novel variants provides a foundation for functional studies, assessing treatment response, developing targeted therapies, and decoding the molecular mechanisms underlying this complex disorder.

LHX3 is a LIM-homeodomain transcription factor gene located on chromosome 9q34.3 and is a member of the LIM-homeodomain family. It plays a critical role in pituitary gland development, the specification of anterior pituitary cell lineages, and the development of the neuroendocrine and auditory systems [4]. In addition, LHX3 plays a critical role in spinal cord and motor neuron development during embryogenesis. LHX3-related CPHD follows an autosomal recessive inheritance pattern. The clinical manifestations of LHX3 variants include deficiencies of growth hormone (GH), thyroid-stimulating hormone (TSH), prolactin (PRL), luteinizing hormone (LH), follicle-stimulating hormone (FSH), and, rarely, adrenocorticotropic hormone (ACTH). Notable extra-pituitary manifestations of LHX3 variants include sensorineural hearing loss and limited neck rotation [5]. To the best of our knowledge, the number of reported LHX3 variants has increased substantially over time. A previous case report published in 2017 described 16 variants; a total of 32 pathogenic and likely pathogenic variants are currently cataloged in the Human Gene Mutation Database (HGMD), reflecting the expanding genotypic spectrum of LHX3-associated disorders [6]. Here, we described two siblings of CPHD who presented with severe short stature and limited neck movements. In both, molecular genetic analysis revealed a similar homozygous novel in-frame deletion in LHX3, c.634_636del (p.Glu212del), and the parents were found to be heterozygous carriers. We did not identify any additional genetic variants that could contribute to CPHD in these siblings.

## Case presentation

The index case (elder male sibling) presented at 10 years of age with profound growth retardation. He was born to parents with a third-degree consanguineous marriage, delivered by elective lower-segment cesarean section (LSCS), with a birth weight of 2.4 kg (Figure 1).

**Figure 1:**
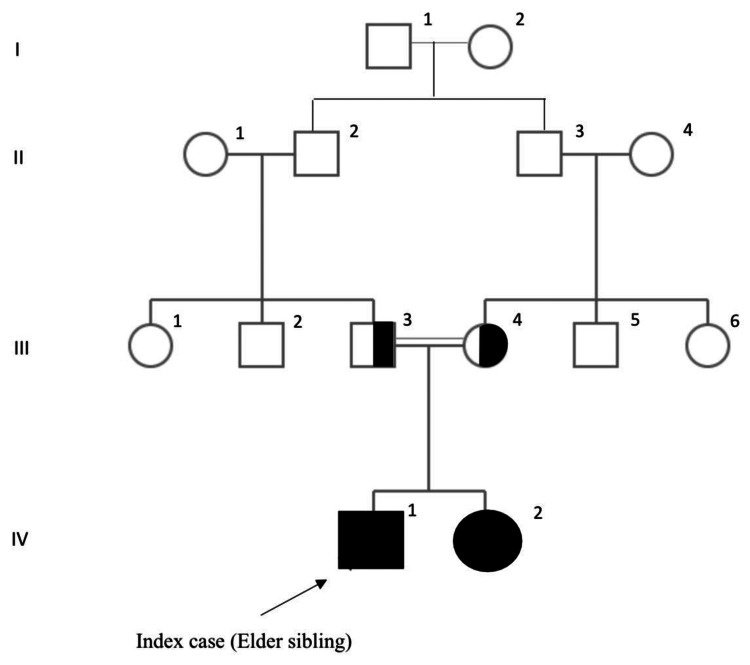
Pedigree showing the affected family. A double line indicates a consanguineous marriage; solid symbols designate siblings with homozygous LHX3 mutations; and both parents are represented by half-solid symbols, indicating heterozygous asymptomatic carriers.

During the early neonatal period, he developed recurrent episodes of hypoglycemia and had bilateral cryptorchidism. He was diagnosed with central hypothyroidism, central hypocortisolism, and hypogonadism suggestive of CPHD. Oral hydrocortisone and levothyroxine replacement therapy were initiated on the 15th day of life, and the subsequent infantile period was uneventful. At two years of age, he underwent bilateral orchidopexy.

On examination, he had a height of 95 cm (height standard deviation score {SDS}: -6.8) with dysmorphic facial features, such as frontal bossing, midfacial hypoplasia, and a depressed nasal bridge, which were observed. Additionally, he had a short neck with a height-neck ratio of 14:1, restricted neck rotation to 90^◦^, and brachymetatarsia of the left 4th toe. Mild hypotonia was present, and examination of the external genitalia revealed micropenis (stretched penile length, 2 cm) and small intrascrotal testes (1.5 mL each) (Figures 2A-2D).

**Figure 2:**
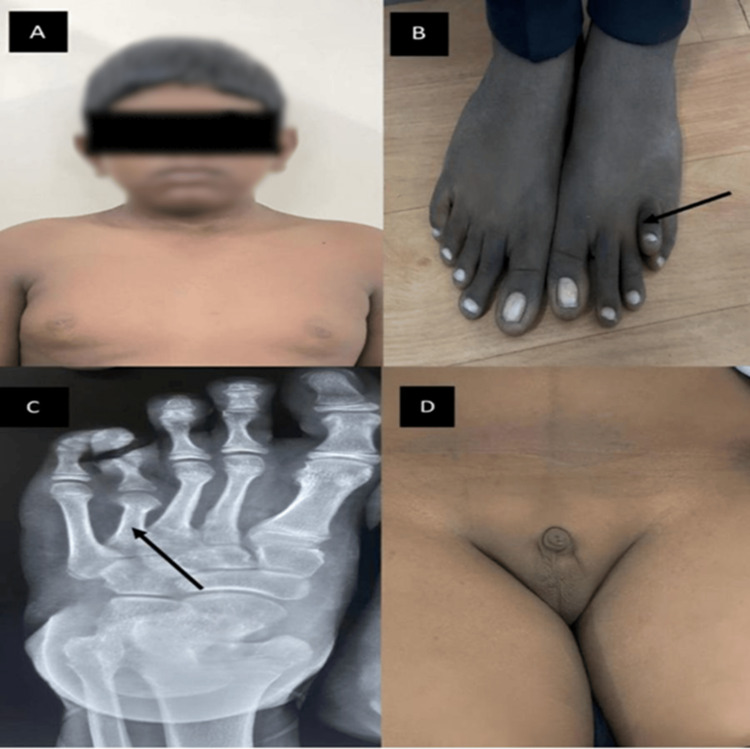
Clinical phenotype of the affected siblings. (A) Dysmorphic facies (elder sibling). (B) Clinical photograph demonstrating brachymetatarsia. (C) Foot radiograph showing brachymetatarsia (arrow). (D) Clinical photograph of infantile external genitalia with a hypoplastic scrotum after orchidopexy.

He had a bone age of five years and a height age of four years. MRI of the brain showed a hypoplastic anterior pituitary. MRI of the cervical spine showed basilar impression of the dens of the axis along with a short clivus and mild cervical scoliosis (Figures 3A, 3B).

**Figure 3:**
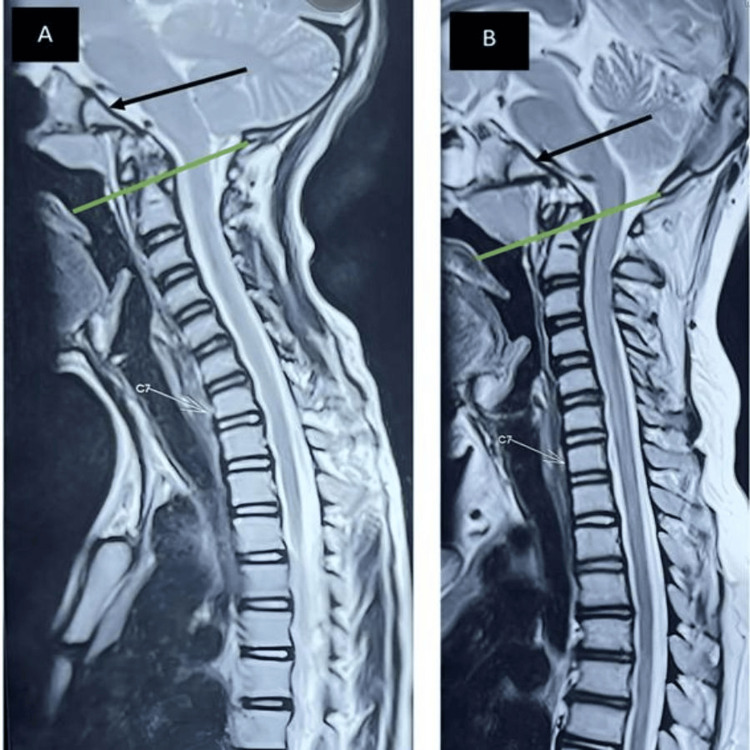
T2 sagittal MRI sections of cervical spine in both siblings showing basilar invagination, as indicated by violation of the chamberlain line (green line) by the dens axis more than 3 mm and short clivus (black arrow).

On psychological assessment, he had a subnormal intelligence quotient (IQ) of 71. The audiometric examination was within normal limits. After priming with 2 mg of estradiol valerate for three days, he underwent provocative testing with clonidine and glucagon at 48-h intervals, and growth hormone (GH) deficiency was confirmed (peak GH: 0.08 ng/mL; normal response: >10 ng/mL). Treatment with rhGH was instituted at a dose of 0.24 mg/kg/week along with levothyroxine and hydrocortisone replacement. The index case showed an excellent growth spurt of 13 cm in the first year of treatment. After seven years of rhGH, his overall growth rate improved, with the height SDS changing from -6.8 to -2.93 and absolute height increasing from 95 cm to 150 cm. At the age of 17 years, his gonadotropin levels were very low, consistent with prepubertal status on leuprolide-stimulation testing. Injection HCG 2000 U thrice weekly was initiated along with recombinant follicle-stimulating hormone (FSH) 75 IU on alternate days for pubertal induction.

His younger sister presented at 8.6 years of age with severe short stature and an apparent short neck with restricted rotation in a similar fashion. Her neonatal and infantile periods were uneventful. She was 80 cm tall (SDS for height -8.5) and had a bone age of four years. No dysmorphic facial features were present on examination. She also had similar multiple pituitary hormonal deficits identical to her brother (Table 1). MRI showed a hypoplastic pituitary (4 mm in height, 100 mm^3^ in volume). On replacement with rhGH, hydrocortisone, and levothyroxine, the patient showed rapid catch-up growth, gaining 12 cm in one year. Puberty was induced at 14 years with the tablet estradiol valerate 0.25 mg with gradually increasing doses. Clinical, auxological, radiological characteristics, and hormonal parameters of both siblings at presentation are summarized in Table 1.

**Table 1:** Clinical, auxological, radiological, and baseline hormonal characteristics of both siblings. SDS: standard deviation score; BA/CA ratio: bone age/chronological age ratio; ACTH: adrenocorticotropic hormone; TSH: thyroid-stimulating hormone; FT4: free T4; IGF-1: insulin-like growth factor 1; GH: growth hormone; LH: luteinizing hormone; FSH: follicle-stimulating hormone; SNHL: sensorineural hearing loss

Variables	Sibling 1	Sibling 2
Clinical and auxological parameters		
Age at presentation (years)	10	8.6
Birth weight (kg)	2.4	2.3
Height (cm)	95	80
Height SDS	-6.8	-8.6
Bone age (years)	5	4
BA/CA ratio	0.50	0.46
Neonatal manifestations	Hypoglycemia, cryptorchidism	-
Extra-pituitary abnormalities	Hypotonia, facial dysmorphism, neck rigidity, brachymetatarsia, and intellectual disability	Neck rigidity
SNHL	-	-
Radiological characteristics		
Pituitary morphology (MRI)	Anterior pituitary hypoplasia	Anterior pituitary hypoplasia
Other radiological abnormalities	Basilar invagination, short clivus, and cervical scoliosis	Basilar invagination, short clivus, and cervical scoliosis
Hormonal profile		
Cortisol (6.27-18) µg/dL	1.8	2.06
ACTH (<46.00) pg/mL	6	8
TSH (0.3-5.5) mIU/mL	0.09	0.23
FT4 (0.89-1.72) ng/dL	0.78	0.81
IGF-1 (60-265) ng/mL	25	18.2
Peak GH ng/mL (>10; clonidine stimulation test) 60 min	0.03	0.01
Peak GH ng/mL (>10; clonidine stimulation test) 90 min	0.08	0.03
LH (2.4-12.6) mIU/mL	0.210	0.132
FSH (3.5-12.5) mIU/mL	1.45	2.1
Prolactin (2-25) ng/mL	12	17

Considering the co-occurrence of CPHD in both siblings in the presence of parental consanguinity with a background of extra-pituitary manifestations like intellectual disability and restricted neck movements, whole-exome sequencing was done for both siblings as well as parents to find out the genetic etiology.

Genetic analysis

Genomic DNA was extracted from peripheral blood using the Qiagen DNA Blood Kit. Library preparation was performed, and sequencing was carried out on the Illumina platform with a mean coverage depth of >80-100×. Variant analysis was performed according to the Genome Analysis Toolkit (GATK) best-practices framework (Cambridge, UK: Broad Institute) using the Sentieon pipeline (Mountain View, CA: Sentieon Inc.) for germline variant identification. Sequencing reads were aligned to the human reference genome (GRCh38) using the Burrows-Wheeler Aligner (BWA) [PMID: 20080505] (Cambridge, UK: Broad Institute). Subsequent processing steps, including duplicate removal, base quality recalibration, and indel realignment, were carried out using Sentieon tools (Mountain View, CA: Sentieon Inc.). Variant calling was performed using the Sentieon Haplotype Caller (Mountain View, CA: Sentieon Inc.). Identified germline variants were comprehensively annotated using the VariMAT pipeline. Gene annotation was performed using the Variant Effect Predictor (VEP) [PMID: 20562413] against the Ensembl Release 104 human gene model [PMID: 34791404]. In addition to single-nucleotide variants (SNVs) and small insertions/deletions (indels), copy number variants (CNVs) were detected from targeted sequencing data using the ExomeDepth algorithm [PMID: 22942019], which identifies CNVs by comparing read depths of the test sample against matched aggregate reference datasets. Clinically relevant variants in coding and non-coding regions were annotated using published literature and curated disease databases, including ClinVar, OMIM, Human Gene Mutation Database (HGMD), LOVD, DECIPHER, and SwissVar. Common population variants were filtered based on allele frequency data from major population databases, including 1000 Genomes, gnomAD, dbSNP, and other ancestry-specific reference datasets. The pathogenicity of the identified in-frame deletion variant (p.Glu212del) was evaluated using in silico prediction tools optimized for insertion/deletion variants, including MutationTaster2 (Charité - Universitätsmedizin Berlin: Berlin, Germany) and PROVEAN (La Jolla, CA: J. Craig Venter Institute). The computational evidence obtained was incorporated into the ACMG classification, including PP3 supporting evidence where applicable.

A novel homozygous 3-base pair deletion in exon 5 of the LHX3 gene (RefSeq transcript NM_178138.6, GRCh38 version) that results in the in-frame deletion of amino acids (p.Glu212del) was detected in both (Figure 4).

**Figure 4:**
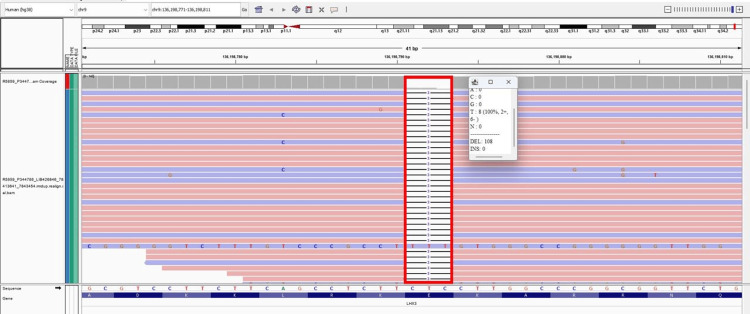
Variant visualization using Integrative Genomics Viewer (IGV) of next-generation sequencing (NGS) data in exon 5 of the LHX3 gene showing the in-frame deletion c.634_636del (p.Glu212del), resulting from the deletion of the glutamic acid residue at position 212. The red box indicates the homozygous deletion variant, which is present in all mapped reads within the affected exonic region. Integrative Genomics Viewer (IGV) (Cambridge, UK: Broad Institute)

Based on standard variant interpretation criteria, this variant is rare and absent from common population databases, including the 1000 Genomes Project and gnomAD, supporting classification under the PM2 criterion [7]. The variant has an allele frequency of 0 across the available population datasets, supporting its rarity and fulfilling the ACMG PM2 criterion. The in silico prediction of the variant is damaging across multiple computational tools, including MutationTaster2 and PROVEAN. The computational evidence obtained was incorporated into ACMG classification, including PP3 supporting evidence where applicable (PP3) [7]. The same variant was identified in the homozygous state in the affected siblings and suggests that the variant may be co-segregating with disease phenotype (PP1), and also the OMIM-described gene LHX3 (OMIM No:600577), implicated in combined pituitary deficiency 3 (OMIM No:221730), matches the patient’s phenotype (PP4) [7]. Both parents are unaffected, and Sanger sequencing revealed the parents to be heterozygous carriers (Figure 5).

**Figure 5:**
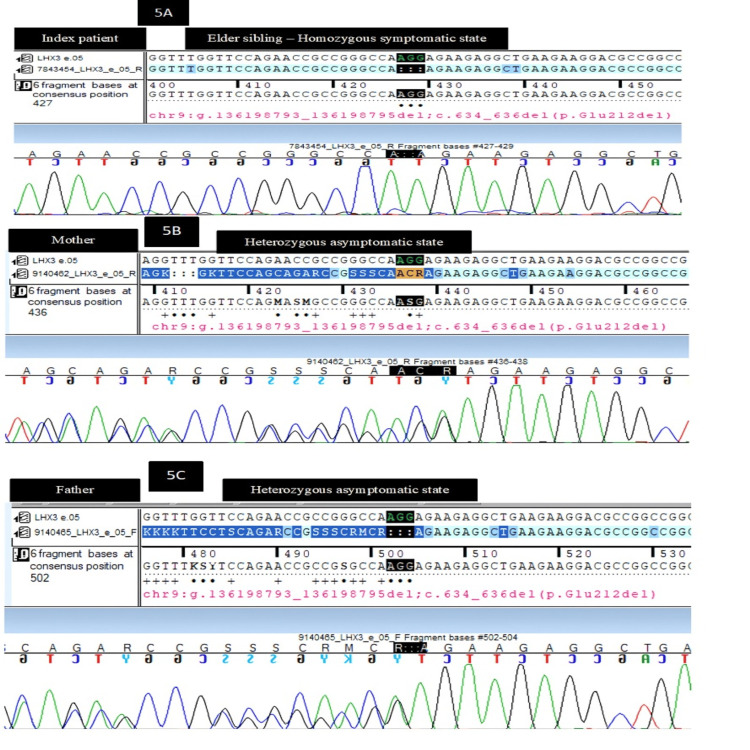
Sanger sequencing chromatograms showing the c.634_636del (p.Glu212del) in the LHX3 gene. The variant is present in a homozygous state in the index case (A). Both parents (B and C) are asymptomatic heterozygous carriers.

So, as per the ACMG guidelines, we have found moderate and supporting evidence for this variant and have reported it as of uncertain significance for now (PM2, PP3, PP1, PP4) due to a lack of well-established in vitro and in vivo functional studies demonstrating a deleterious effect [7]. However, this variant could be novel and potentially relevant, as two affected siblings in the same family carry a rare biallelic variant, with parents being heterozygous carriers, and it clinically correlates with the gene's phenotype.

## Discussion

CPHD encompasses a broad clinical spectrum ranging from potentially devastating multiple hormone deficiencies in the neonate to insidious-onset gradual presentation in childhood or adolescence [8]. Pituitary stalk interruption syndrome (PSIS) often serves as a key marker of an underlying diagnosis of CPHD in patients with initial presentation of isolated growth hormone deficiency (IGHD), enabling earlier diagnosis and better growth response [9]. Additionally, genetic confirmation strongly correlates with treatment responsiveness in isolated growth hormone deficiency (IGHD) and CPHD cohorts. Recent data from a South Indian cohort of isolated growth hormone deficiency (IGHD) demonstrated a high prevalence of genetic variants, predominantly involving GHRHR mutations, emphasizing the importance of genetic evaluation in children with severe short stature and pituitary hormone deficiencies [10]. The extra-pituitary manifestations of CPHD are often variable and take syndromic form due to mutations involving transcription factors linked with early pituitary development. The most commonly reported extra-pituitary manifestations include optic nerve hypoplasia (HESX1), anophthalmia/microphthalmia (SOX2, OTX2), spinal abnormalities (LHX3), and cerebellar abnormalities (LHX4) [11].

The understanding of the genetic spectrum of combined pituitary hormone deficiency (CPHD) has greatly evolved in recent years with the emergence of dynamic NGS tools. Predictors of mutation positivity include familial cases, associated extra-pituitary manifestations, neuroradiological abnormalities, and the geographical origin of the patient [12].

PROP1 (Prophet of PIT1) and PIT1 (POU1F1) are pituitary-specific transcription factors that play crucial roles in embryonic pituitary development and the differentiation of hormone-producing cells. PROP1 is involved in the early stages of pituitary organogenesis and regulates the development of multiple anterior pituitary cell lineages, whereas PIT1 is essential for the terminal differentiation and function of somatotrophs, lactotrophs, and thyrotrophs. Consequently, pathogenic variants in these genes result in varying degrees of combined pituitary hormone deficiency (CPHD). Overall, the PROP1 mutations are the most commonly reported genetic abnormality in CPHD worldwide. In contrast, PIT1 genetic variants are widely reported in Indian cohorts [13,14]. Data on the longitudinal effects and outcomes of CPHD due to LHX3 variants are limited. To the best of our knowledge, this is the second case report of LHX3 abnormality from the Indian subcontinent [15]. Patients with different LHX3 variants have differences in clinical presentation, hormonal deficits, and varying pituitary morphology [15]. The most frequently reported LHX3-related genetic alterations include missense, nonsense, and gross deletion variants. Some patients with the LHX3 variant exhibit rigid cervical spines with limited neck rotation, possibly linked to its role in motor neuron development. Anatomical abnormalities of the cervical spine resembling articulation abnormalities have been reported in previous studies [16]. Individuals with LHX3 variants should be closely monitored for cervical spine mobility and the development of scoliosis.

Rajab et al. described four patients with LHX3 variants, with variable degrees of restricted neck rotation and hearing deficit in 2008 [17]. Although most patients develop severe hormone deficiency after birth, some develop a milder form with preserved neck rotation [15]. Profound sensorineural hearing loss (SNHL) is reported with a 23bp gene deletion, and the p.Y116C variant is associated with mild to moderate hearing loss [18]. Similar to our case, hearing loss is less frequently reported with other variants. It is also interesting to note that an enlarged anterior pituitary is reported with a 23bp deletion and p.A210V variants. A less severe endocrine phenotype with growth paralleling the third centile was noted with the W224ter variant [19].

Corticotrope insufficiency is less frequently reported in LHX3 variants. A homozygous stop mutation in exon 2: c.229C>T (CGA > TGA), Arg77stop (R77X), leading to ACTH deficiency was described by Bonfig et al. in a 1.5-year-old boy [20]. Recently, Ramzan et al. described three patients from two unrelated families with rigid cervical spine, scoliosis, developmental delay, along with ACTH and other pituitary hormonal deficiencies [6]. It is also noteworthy that our patients also had ACTH deficiency. One of our reported siblings even had a documented episode of adrenal crisis from missing a single daily dose of glucocorticoid therapy. ACTH deficiency is increasingly reported in recent studies with novel pathogenic LHX3 variants. Therefore, longitudinal screening of ACTH deficiency is warranted in patients with LHX3 mutations.

Here, we described two Indian siblings born of a consanguineous background with a novel homozygous in-frame deletion variant of the LHX3 gene: (RefSeq transcript NM_178138.6) c.634_636del (p.Glu212del), with both parents being heterozygous carriers. This is consistent with the clinical phenotype of previously reported genetic variants. The variant region is conserved across species and falls in the DNA-binding homeodomain of the LHX3 transcription factor. Additionally, both parents had normal hormonal parameters on evaluation, consistent with asymptomatic heterozygous carriage of the LHX3 variant.

The three-base pair deletion, 634_636del (p.Glu212del), represents a novel in-frame deletion. This type of deletion maintains the reading frame of the LHX3 gene, resulting in the deletion of a single amino acid (glutamic acid at position 212). Although in-frame deletions are less frequent than other types, according to theoretical models, this variant could potentially disrupt protein-protein interactions, DNA binding, and protein stability, ultimately affecting the specification of pituitary cell types. Similar to the variant location found in our case (p.Glu212del, exon 5, LHX3), Pfaeffle et al., also described a biallelic variant (p.Ala210Val, exon 5, LHX3) in two siblings born of consanguineous Indian parents and the mutation resulted in impaired DNA binding and transcriptional activation in pituitary cells, which resulted in a similar degree of hormonal deficiencies to those observed in our patients [15]. Since the identified variant location (exon 5) is observed to be near the previously reported DNA-binding domain variant, the functionality of the LHX3 transcription factor may be similarly affected and clinically correlated with the affected siblings. While detailed functional studies were not performed, both affected siblings were found to carry the variant in the homozygous state, whereas the parents were heterozygous carriers. This pattern suggests that the variant may co-segregate with the disease-causing phenotype within the family.

Considering the consanguineous background, the presence of the same novel variant in both affected siblings with a similar phenotype, heterozygous carrier status in the unaffected parents, and supportive in silico predictions provide evidence consistent with a possible association between this LHX3 variant and the observed phenotype. Additional sequencing analysis did not identify other clinically relevant variants that could readily explain the presentation. Although functional studies were not performed because of financial constraints, future in vitro and in vivo studies are required to establish the biological significance and pathogenicity of this variant. Reporting such rare variants from consanguineous populations contributes to expanding the phenotypic and genotypic spectrum of LHX3-associated disorders and may facilitate future genotype-phenotype correlations and genetic counseling.

## Conclusions

The phenotypic presentation of short stature, short neck, and limited neck movements with hypopituitarism warrants genetic testing for LHX3 variants. Identifying an LHX3 mutation at an early stage helps with auditory evaluation and monitoring of the cervical spine and neurodevelopmental abnormalities. Continual monitoring for ACTH deficiency is necessary, as it is increasingly reported in recent studies involving LHX3 variants. This study contributes to the growing body of knowledge on LHX3 variants by identifying a novel in-frame LHX3 variant. This highlights the need for future functional studies to understand the molecular mechanisms of novel variants. Reporting of novel mutations across diverse populations expands our knowledge and global perspective on CPHD.
